# Tirofiban for Preventing Early Neurological Deterioration in Acute Ischemic Stroke Within 48 Hours of Onset: Evidence From a Dual‐Method Analysis Using Propensity Score Matching and Multivariable Regression

**DOI:** 10.1002/cns.70718

**Published:** 2025-12-28

**Authors:** Qianru Wen, Ying Zhao, Yongbing Deng, Shaobin Guan, Siqi Zhang, Jieming Zhou, Pengtao Zhu, Xuejiao Fan, Junjie Li, Yang Chen, Guoqing Cai, Yihong Huang, Shugong Zheng, Heng Meng

**Affiliations:** ^1^ Department of Neurology The Sixth Affiliated Hospital of Jinan University Dongguan China; ^2^ Dongguan Key Laboratory of Central Nervous System Injury and Repair Dongguan China; ^3^ Department of Neurology The First Affiliated Hospital of Jinan University Guangzhou China; ^4^ Department of Neurology Dongguan Houjie Hospital Dongguan China; ^5^ Department of Neurology Dongguan Dalang Hospital Dongguan China

**Keywords:** acute ischemic stroke, early neurological deterioration, multivariable regression, non‐large vessel occlusion, propensity score matching, Tirofiban

## Abstract

**Background:**

Previous studies have indicated the potential benefits of tirofiban in preventing early neurological deterioration (END) in acute ischemic stroke (AIS) within 24 h of symptom onset. However, its efficacy and safety over a broader time window require further evaluation.

**Methods:**

This multicenter study analyzed prospective data from AIS patients without large vessel occlusion (LVO), enrolled within 48 h of onset and with baseline NIHSS scores of 4–15. Participants received either intravenous tirofiban or oral antiplatelet therapy. The primary efficacy endpoint was the occurrence of END (increase in NIHSS score ≥ 2 points within 7 days). The primary safety endpoint was intracranial hemorrhage within 90 days. The study employed a combined analysis method of multivariable regression and propensity score matching (PSM).

**Results:**

Among 371 enrolled patients (198 in the tirofiban group, 173 in the oral antiplatelet group), compared with the oral antiplatelet group, the incidence of END in the tirofiban group was significantly lower, as indicated by multivariate regression analysis (9.6% vs. 18.0%, *p* = 0.038) and PSM (10.6% vs. 19.7%, *p* = 0.031). Both statistical methods indicated that intravenous tirofiban can significantly facilitate early neurological improvement in patients at 7 and 14 days (*p* < 0.05) and enhance the probability of a mRS score of 0–2 at 90 days (*p* < 0.05). Subgroup analysis indicated particular benefit for patients with branch atheromatous disease (BAD) (*p* = 0.041). No symptomatic intracranial hemorrhage occurred in either group.

**Conclusion:**

For AIS patients without LVO, early intravenous tirofiban within 48 h of onset effectively reduced the risk of END and promoted early or long‐term neurological improvement without increasing bleeding risk, suggesting a potential therapeutic benefit in an extended time window, especially for the BAD subtype.

**Trail Registration:**

Chinese Clinical Trial Registry (chictr.org.cn): ChiCTR2200061110.

## Graphical abstract

Previous studies have shown that tirofiban may reduce the risk of early neurological deterioration within 24 h after symptom onset in patients with acute ischemic stroke (AIS). However, evidence on its efficacy beyond this time window is limited. This study suggests that tirofiban may still confer potential therapeutic benefits in AIS patients without large vessel occlusion even when administered within an extended time window.

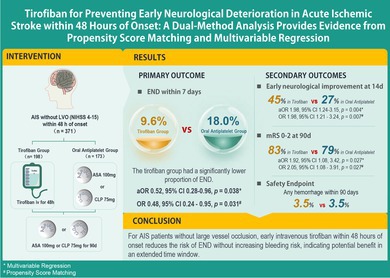

## 1 Introduction

The occurrence of early neurological deterioration (END) in patients with acute ischemic stroke (AIS) presents a major challenge in clinical management. Approximately 26% to 43% of AIS patients experience END within the first week [1]. Even when intensive care is provided in comprehensive stroke units, the incidence of END remains at 8% to 18% among AIS patients [2, 3]. More importantly, END has been strongly linked to poor clinical outcomes.

Reperfusion therapy, including endovascular treatment (EVT) and intravenous thrombolysis (IVT), is widely regarded as the most effective strategy for early management of AIS. However, its applicability is restricted to a limited number of patients due to the narrow therapeutic time window, multiple contraindications, and technical limitations [4]. For patients who do not qualify for reperfusion therapy, clinical guidelines recommend standard treatment with aspirin and clopidogrel, either as monotherapy or in combination [5]. Nevertheless, despite receiving conventional antiplatelet therapy, some patients still develop END.

Tirofiban, a highly selective inhibitor of platelet glycoprotein (GP) IIb/IIIa receptors with a rapid onset and short half‐life, exerts reversible inhibition of platelet aggregation [6, 7]. Tirofiban has demonstrated efficacy in reducing the risk of thrombosis‐related complications [8, 9, 10, 11]. Furthermore, accumulating clinical evidence supports the effectiveness of tirofiban, either alone or in combination with either IVT or EVT, in managing progressive stroke. It exhibits favorable safety and efficacy in promoting vascular recanalization and improving long‐term functional prognosis [12, 13, 14, 15, 16, 17, 18]. Recent findings from the TREND study indicate that in patients with non‐cardioembolic stroke presenting within 24 h of symptom onset, tirofiban reduces the risk of END without increasing the risk of bleeding [19]. However, a considerable proportion of patients do not arrive at the hospital within 24 h after symptom onset to receive timely treatment. For those who exceed this time window, the clinical benefits of tirofiban administration remain uncertain. Thus, further investigation into the efficacy of tirofiban in a broader patient population is warranted.

We conducted the present study to test the hypothesis that early intravenous administration of tirofiban could prevent END in AIS patients without LVO within 48 h of symptom onset and did not increase the risk of bleeding.

## 2 Method

### 2.1 Study Design

This study is a prospective, multicenter cohort study from four stroke centers in southern China. The research protocol was reviewed and approved by the ethics committee of the Sixth Affiliated Hospital of Jinan University (MEC [2022] 021). All clinical and laboratory procedures were conducted in accordance with the ethical principles outlined in the Declaration of Helsinki. Written informed consent was obtained from each participant or their legally authorized representative prior to enrollment.

### 2.2 Participants

The inclusion criteria were as follows: (1) adults aged 18 years or older; (2) met the diagnostic criteria for AIS [5]; (3) onset of symptoms or last known normal within 48 h; (4) NIHSS score of 4 to 15 points. The exclusion criteria included: (1) evidence of large vessel occlusion (LVO) on CT or MRI; (2) pre‐existing disability with a pre‐stroke modified Rankin scale (mRS) score ≥ 2; (3) planned to receive EVT or IVT; (4) cardioembolic ischemic stroke; (5) systolic blood pressure > 180 mmHg or diastolic blood pressure > 110 mmHg despite aggressive treatment; (6) active bleeding disorder, including coagulation abnormalities (platelet count < 100 × 10^9^/L, INR > 1.7), or treatment with a direct oral anticoagulant within the preceding 48 h; (7) severe comorbidities such as malignant tumor, severe infections, or severe renal dysfunction; (8) concurrent use of other anticoagulant medications; (9) history of intracerebral hemorrhage.

### 2.3 Interventions

All AIS patients included in the study received treatment within 48 h of symptom onset. Patients were stratified into two groups: the intravenous tirofiban group and the oral antiplatelet group, based on the preference of the patient as well as their family and the decision of the physician. In the tirofiban group, intravenous tirofiban was administered with an initial infusion of 0.4 μg/kg/min for 30 min, followed by a maintenance infusion of 0.1 μg/kg/min for up to 48 h [20]. Subsequently, all patients were prescribed oral antiplatelet therapy, including either aspirin 100 mg or clopidogrel 75 mg. A 4‐h overlap period between intravenous tirofiban and oral antiplatelet therapy was required before discontinuation of the intravenous infusion. In the oral antiplatelet group, patients received either aspirin 100 mg or clopidogrel 75 mg daily for up to 90 days after enrollment. Thereafter, all patients were managed in accordance with the international and Chinese guidelines [5, 21, 22].

### 2.4 Endpoints

The primary efficacy endpoint was the proportion of patients experiencing END in hospital within 7 days after admission. END was defined as an increase of two or more NIHSS points and a minimum one‐point increment in motor power during the first 7 days following admission [23, 24]. The secondary efficacy endpoints included the proportion of early NIHSS improvement at 7 and 14 days, as well as excellent (mRS 0 to 1) and favorable (mRS 0 to 2) functional outcomes at 90 days. Early neurological improvement was defined as neurological recovery to a NIHSS score of 0 or a decrease in NIHSS score of ≥ 4 at discharge, compared with the baseline NIHSS score [25]. The safety endpoints included symptomatic intracranial hemorrhage (sICH) within 72 h, stroke recurrence, and any hemorrhage event within 90 days. sICH was defined as the presence of bleeding on a head CT scan accompanied by clinically significant neurological deterioration (an increase in NIHSS score of ≥ 4 points compared with the baseline) as determined by the clinical investigator [26]. All patients were followed up for 90 days.

### 2.5 Statistical Analysis

The sample size calculation was based on previous trials. We assumed an incidence of END in Chinese patients to be 25.4% [27], with an expected absolute difference of 12 percentage points between the intravenous tirofiban and oral antiplatelet groups. According to our calculations, a sample size of 310 patients (155 per group) would provide 80% power to detect the expected treatment effect at a two‐sided significance level of 0.05. To account for an estimated 10% nonadherence and dropout rates, we aimed to enroll a minimum of 344 patients.

For binary outcome variables, univariate logistic regression analyses were conducted to assess each outcome indicator. Variables with a *p*‐value < 0.05 were subsequently included in the multivariate logistic regression model to adjust for potential confounding factors. Odds ratios (ORs) and corresponding 95% confidence intervals (CIs) were calculated. Propensity score matching was subsequently performed using the nearest neighbor matching method without replacement, with a caliper distance of 0.1, to achieve 1:1 pair matching between patients in the tirofiban group and those in the oral antiplatelet group. After matching, the standardized mean difference (SMD) was calculated to evaluate the balance of baseline characteristics between the two groups; an SMD < 0.1 was considered to indicate adequate covariate balance. In the matched cohort, all efficacy and safety endpoint outcomes were reassessed using conditional logistic regression, and results were reported as ORs with 95% CIs.

A subgroup analysis of the primary outcome was performed across five prespecified subgroups stratified by age (≤ 65 years or > 65 years), gender (female or male), duration of onset (≤ 24 h or 24–48 h), presence of large‐artery atherosclerosis (yes or no), and branch atheromatous disease (BAD) (yes or no). The diagnosis of BAD was based on the Chinese Expert Consensus on BAD [28]. BAD primarily involves AIS affecting the territories supplied by the lenticulostriate artery (LSA) and the paramedian pontine artery (PPA). The diagnostic criteria require the presence of infarcts in these specific vascular territories on diffusion‐weighted imaging (DWI), involving at least three transverse layers. The PPA supplies the ventral surface of the pons and does not cross the midline. Exclusion criteria include imaging evidence of ≥ 50% stenosis in a major supplying artery, presence of unstable plaques suggestive of artery‐to‐artery embolism, or DWI findings indicative of cortical infarction, watershed infarction, or multiple cerebral infarctions.

Statistical analyses were conducted using R software (version 4.1.0), SPSS software (version 26), and MSTATA software (www.mstata.com). A *P*‐value of less than 0.05 was considered statistically significant for all endpoints, indicating a significant difference between the two groups.

## 3 Results

**FIGURE 1:**
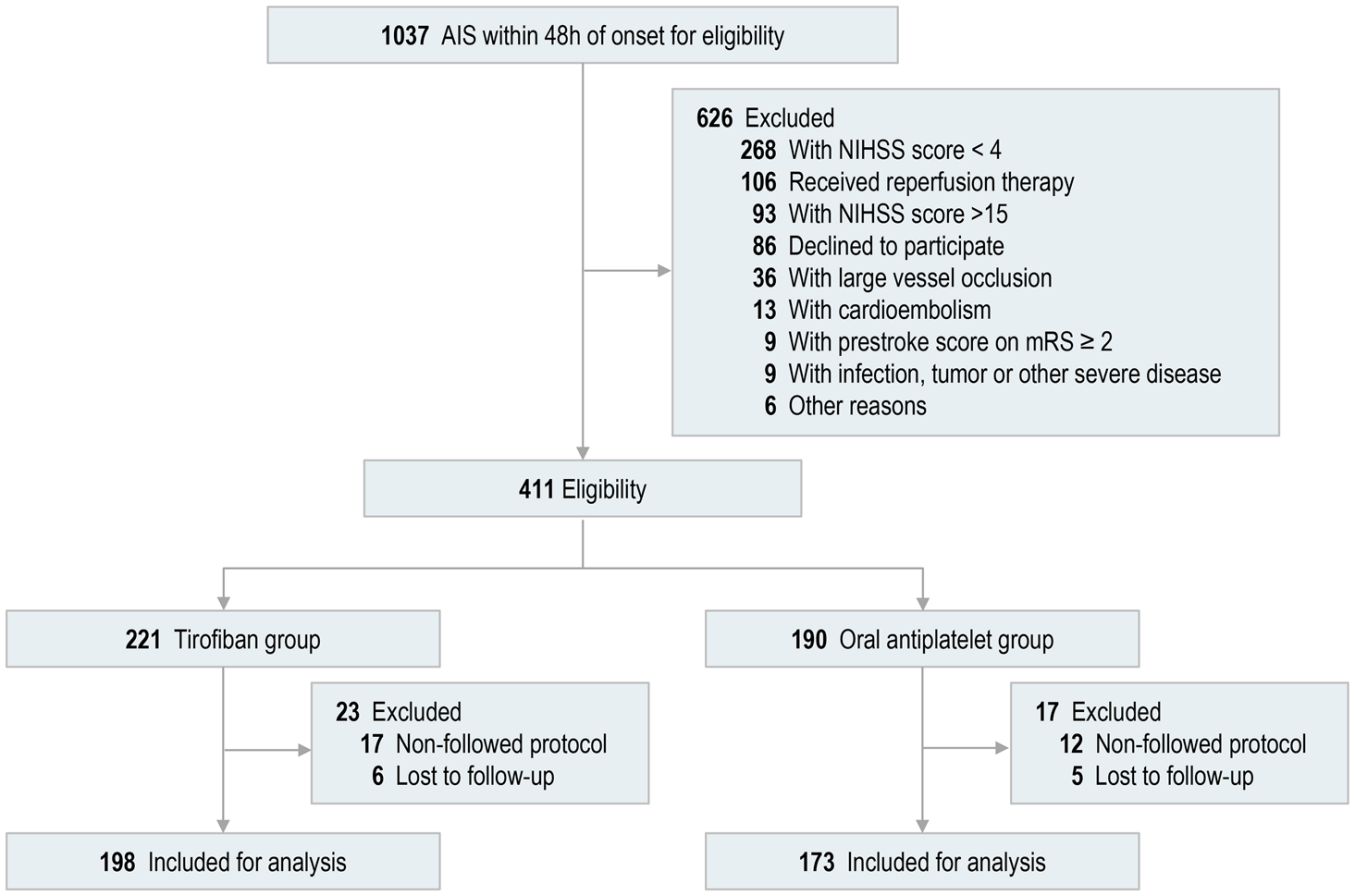
Flow diagram of the participants selection. AIS, acute ischemic stroke; NIHSS, National Institutes of Health Stroke Scale; mRS, modified Rankin scale.

Between January 2022 and June 2024, a total of 1037 patients were screened across four centers in southern China, of whom 626 did not meet the eligibility criteria (Figure 1). A total of 411 patients were enrolled in the study, comprising 221 (53.4%) in the tirofiban group and 190 (46.6%) in the oral antiplatelet group. There were 17 patients in the tirofiban group and 12 patients in the oral antiplatelet group were excluded for violating the research protocol. In addition, six patients in the tirofiban group and five patients in the oral antiplatelet group were lost to follow‐up. Ultimately, 398 patients (198 in the tirofiban group and 173 in the oral antiplatelet group) were included in the analysis.

### 3.1 Baseline Characteristics

In this study, 262 (71.6%) of the participants were male, and 109 (29.4%) were female, with a mean (standard deviation [SD]) age of 60 (11) years. A total of 199 (53.6%) patients were classified as BAD. The mean (SD) NIHSS score at admission was 5.77 (2.01) in the tirofiban group and 5.06 (1.58) in the oral antiplatelet group. When compared with the oral antiplatelet group, the tirofiban group consisted of younger patients and had a higher proportion of patients who had previously used antiplatelet medications. The baseline characteristics of the two groups are summarized in Table 1.

**TABLE 1:** Demographic and clinical characteristics of the patients at baseline.

Characteristics	Overall (*n* = 371)	Tirofiban (*n* = 198)	Oral antiplatelet (*n* = 173)	*p*
Female, *n* (%)	109 (29.4)	55 (27.8)	54 (31.2)	0.469
Age (years), Mean (SD)	60 (11)	59 (11)	62 (11)	0.006
Medical history, *n* (%)				
Hypertension	244 (65.8)	125 (63.1)	119 (68.8)	0.252
Diabetes mellitus	98 (26.4)	46 (23.2)	52 (30.1)	0.137
Previous stroke	70 (18.9)	31 (15.7)	39 (22.5)	0.091
Previous antiplatelet drug	53 (14.3)	19 (9.6)	34 (19.7)	0.006
Smoking	86 (23.2)	42 (21.2)	44 (25.4)	0.336
Drinking	45 (12.1)	21 (10.6)	24 (13.9)	0.336
Clinical data, Mean (SD)				
Admission‐NIHSS	5.44 (1.85)	5.77 (2.01)	5.06 (1.58)	< 0.001
TOAST classification, *n* (%)				
Large‐artery atherosclerosis	124 (33.4)	66 (33.3)	58 (33.5)	0.955
Small‐vessel occlusion	234 (63.1)	126 (63.6)	108 (62.4)	
Undetermined	6 (1.6)	3 (1.5)	3 (1.7)	
Other determined	7 (1.9)	3 (1.5)	4 (2.3)	
Duration of onset, *n* (%)				
≤ 24 h	258 (69.5)	144 (72.7)	114 (65.9)	0.154
24‐48 h	113 (30.5)	54 (27.3)	59 (34.1)	
BAD, *n* (%)	199 (53.6)	109 (55.1)	89 (51.4)	0.487

Abbreviations: BAD, branch atheromatous disease; mRS, Modified Rankin Score; NIHSS, National Institute of Health Strock Scale; SD, standard deviation; TOAST, trial of ORG 10172 in acute stroke treatment.

PSM resulted in 142 matched pairs with balanced baseline characteristics. The comparison of baseline characteristics between the tirofiban group and the oral antiplatelet group after PSM is presented in Table 2. After matching, the SMDs decreased significantly, with SMD < 0.1 after matching.

**TABLE 2:** Baseline characteristics of groups (after propensity score matching).

Variables	Before matching			After matching		
	Tirofiban (*N* = 198)	Oral antiplatelet (*N* = 173)	SMD^△^	Tirofiban (*n* = 142)	Oral antiplatelet (*n* = 142)	SMD^△^
Demography						
Female, *n* (%)	55 (27.8)	54 (31.2)	0.074	39 (27.5)	44 (31.0)	0.076
Age (year), Mean (SD)	58.5 (11.0)	61.7 (11.3)	0.284	60.1 (10.6)	60.8 (11.3)	0.058
Medical history, *n* (%)						
Hypertension	125 (63.1)	119 (68.8)	0.122	92 (64.8)	92 (64.8)	0.000
Diabetes mellitus	46 (23.2)	52 (30.1)	0.149	39 (27.5)	40 (28.2)	0.015
Previous stroke	31 (15.7)	39 (22.5)	0.165	21 (14.8)	21 (14.8)	0.000
Previous antiplatelet drug	19 (9.6)	34 (19.7)	0.253	16 (11.3)	17 (12.0)	0.018
Smoking	42 (21.2)	44 (25.4)	0.097	35 (24.6)	31 (21.8)	−0.065
Drinking	21 (10.6)	24 (13.9)	0.095	17 (12.0)	15 (10.6)	−0.041
Baseline characteristics						
NIHSS score, Mean (SD)	5.8 (2.0)	5.1 (1.6)	−0.446	5.1 (1.4)	5.2 (1.6)	0.018
TOAST classification, *n* (%)						
Large‐artery atherosclerosis	66 (33.3)	58 (33.5)	0.004	52 (36.6)	47 (33.1)	−0.075
Small‐vessel occlusion	126 (63.6)	108 (62.4)	−0.025	84 (59.2)	89 (62.7)	0.073
Undetermined	3 (1.5)	4 (2.3)	0.053	3 (2.1)	3 (2.1)	0.000
Other determined	3 (1.5)	3 (1.7)	0.017	3 (2.1)	3 (2.1)	0.000
Duration of onset, *n* (%)						
≤ 24 h	54 (27.3)	59 (34.1)	0.144	43 (30.3)	47 (33.1)	0.059
24‐48 h	144 (72.7)	114 (65.9)	−0.144	99 (69.7)	95 (66.9)	−0.059
BAD, *n* (%)	125 (63.1)	104 (60.1)	−0.062	82 (57.7)	86 (60.6)	0.058

Abbreviations: BAD, branch atheromatous disease; mRS, Modified Rankin Score; NIHSS, National Institute of Health Strock Scale; SMD, standardized mean difference; TOAST, trial of ORG 10172 in acute stroke treatment.

### 3.2 Efficacy and Safety Outcomes

#### 3.2.1 Efficacy Analysis

Table 3 presents the efficacy and safety outcomes of multivariable regression and PSM.

**TABLE 3:** Efficacy and safety endpoints.

Endpoints, *n* (%)	Oral antiplatelet (*n* = 173)	Tirofiban (*n* = 198)	Unadjusted		Multivariable‐adjusted analysis		PSM analysis			
			OR (95% CI)	*p*	OR (95% CI)	*p*	Oral antiplatelet (*n* = 142)	Tirofiban (*n* = 142)	OR (95% CI)	*p*
Primary endpoint										
END	32 (18**%**)	19 (9.6**%**)	0.47 (0.25, 0.86)	0.014	0.52 (0.28, 0.96)	0.038^a^	28 (19.7%)	15 (10.6%)	0.48 (0.24, 0.95)	0.031
Secondary endpoints										
NIHSS score improvement										
7 days	20 (12%)	46 (23%)	2.32 (1.31, 4.10)	0.004	2.06 (1.11, 3.81)	0.022^b^	18 (12.7%)	31 (21.8%)	1.92 (1.02, 3.63)	0.041
14 days	46 (27%)	90 (45%)	2.30 (1.48, 3.57)	< 0.001	1.98 (1.24, 3.15)	0.004^c^	40 (28.2%)	62 (43.7%)	1.98 (1.21, 3.24)	0.007
mRS score at 90 days										
0 to 1	96 (55**%**)	125 (63**%**)	1.37 (0.91, 2.08)	0.135	1.68 (1.08, 2.63)	0.022^d^	80 (56.3%)	96 (67.6%)	1.62 (1.00, 2.62)	0.051
0 to 2	136 (79**%**)	164 (83**%**)	1.31 (0.78, 2.20)	0.304	1.92 (1.08, 3.42)	0.027^e^	111 (78.2%)	125 (88.0%)	2.05 (1.08, 3.91)	0.027
Safety endpoints within 90 days										
sICH	0	0	NA	NA	NA	NA	NA	NA	NA	NA
Any hemorrhage	6 (3.5**%**)	7 (3.5**%**)	1.02 (0.34, 3.10)	0.972	1.10 (0.36, 3.41)	0.865^f^	6 (4.2%)	5 (3.5%)	0.83 (0.25, 2.77)	0.758
Stroke recurrence	3 (1.7**%**)	2 (1.0**%**)	0.58 (0.10, 3.50)	0.550	1.73 (0.29, 10.47)	0.546	3 (2.1%)	2 (1.4%)	0.66 (0.11, 4.02)	0.652

Abbreviations: CI, confidence interval; END, early neurological deterioration; mRS, modified Rankin scale; NA, not applicable; NIHSS, National Institutes of Health Stroke Scale; OR, odds ratio; PSM, propensity score matching; sICH, symptomatic intracranial hemorrhage.

^a^ Adjusted for admission NIHSS.

^b^ Adjusted for age, gender, duration of onset, and admission NIHSS.

^c^ Adjusted for admission NIHSS and gender.

^d^ Adjusted for admission NIHSS and diabetes.

^e^ Adjusted for previous stroke, admission‐NIHSS, and duration of onset.

^f^ Adjusted for previous antiplatelet drug.

**FIGURE 2:**
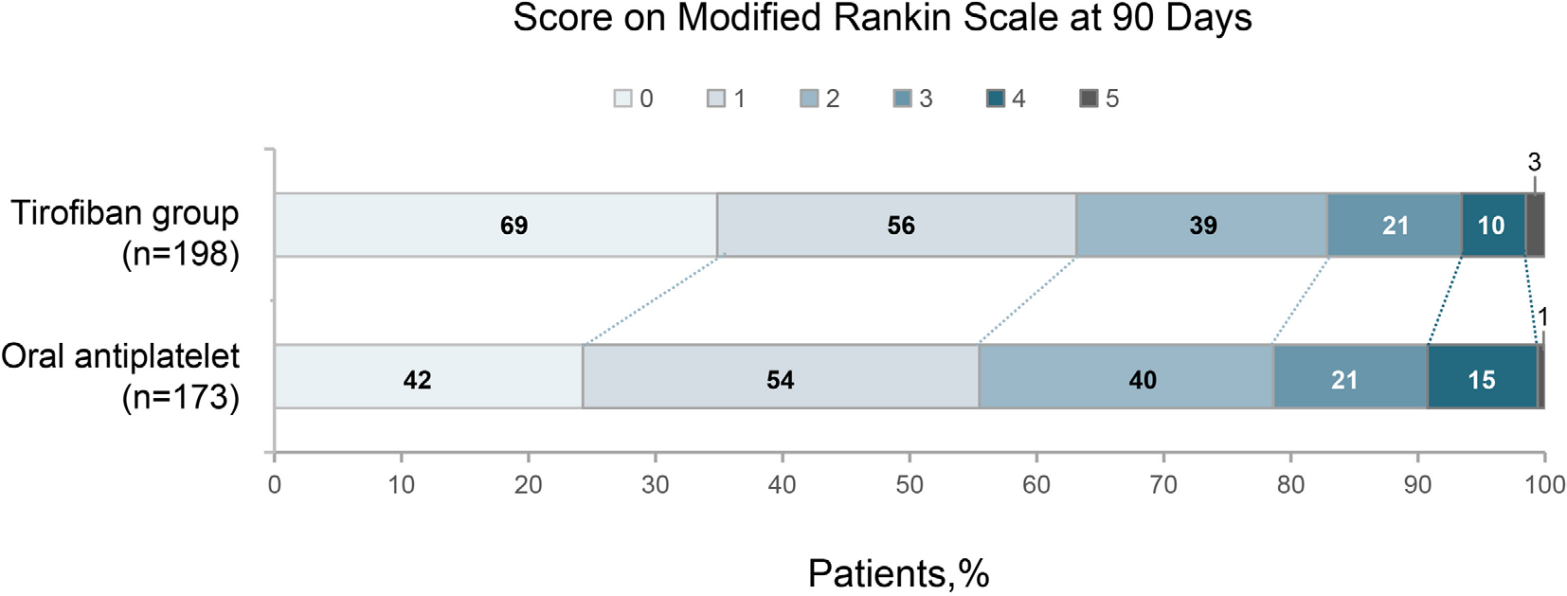
Distribution of scores on the modified Rankin Scale at 90 days.

In multivariable regression, the oral antiplatelet group exhibited a higher incidence of END within 7 days of admission compared to the tirofiban group (18% vs. 9.6%, *p* = 0.038) (Table 3). Tirofiban treatment was associated with significantly improved outcomes in terms of NIHSS score improvement at both 7 days (*p* = 0.027) and 14 days (*p* = 0.004) (Table 3). Additionally, patients receiving tirofiban showed a significantly higher proportion of excellent outcomes (mRS 0 to 1: 55% vs. 63%, *p* = 0.022) and favorable outcomes (mRS 0 to 2: 79% vs. 83%, *p* = 0.027) at 90 days (Figure 2 and Table 3).

After PSM, 10.6% of the patients in the tirofiban group experienced END, whereas 19.7% of the patients in the oral antiplatelet group had END (*p* < 0.05). This difference was statistically significant, aligning with the results of the multivariate regression. Moreover, tirofiban could significantly enhance NIHSS scores of the patients at 7 days and 14 days (*p* < 0.05). In terms of the neurological prognosis at 90 days, tirofiban could significantly elevate the rate of good prognosis (*p* < 0.05). Concerning the excellent prognosis at 90 days, an effective trend was observed for tirofiban (*p* = 0.051), though it did not reach statistical significance.

#### 3.2.2 Safety Analysis

There were no sICH events in either group. The hemorrhage events in both groups were mild and manageable, including upper gastrointestinal bleeding, gingival bleeding, and skin bruising. Before PSM, the hemorrhage incidence rate was 3.5% in each group. Within 90 days, the oral antiplatelet group showed a slightly higher rate of stroke recurrence compared with the tirofiban group (1.7% vs. 1.0%). After PSM, a greater proportion of bleeding risk and stroke recurrence was noted in the oral antiplatelet group. Nevertheless, whether assessed by multivariate regression analysis or the PSM analysis, the differences between the two groups did not attain statistical significance (*p* > 0.05). All safety outcomes are presented in Table 3.

### 3.3 Subgroup Analysis Results

**FIGURE 3:**
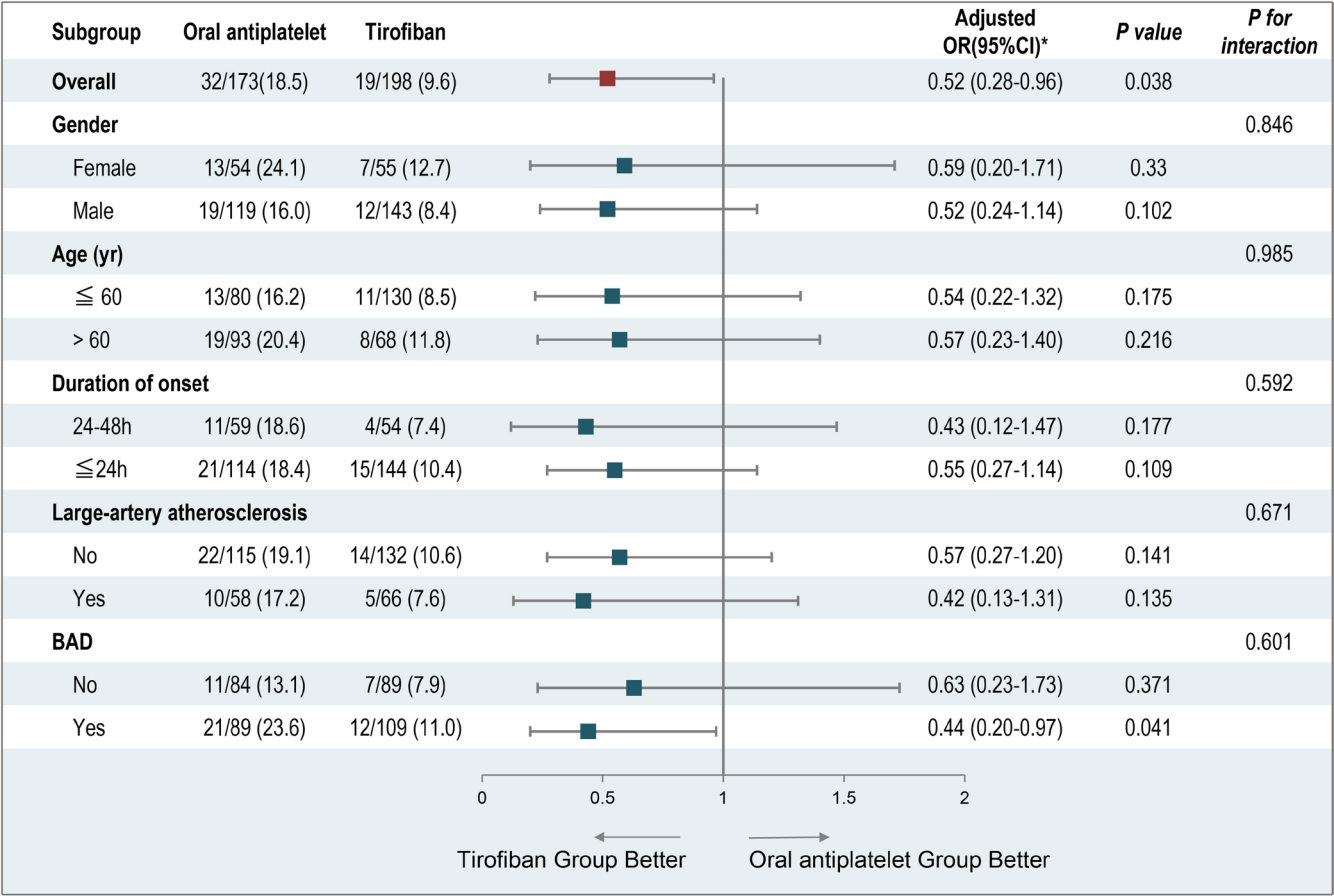
Early neurological deterioration according to the prespecified subgroups. BAD, branch atheromatous disease; CI, confidence interval; OR, odds ratio. *Adjusted: admission NIHSS.

The subgroup analysis of END showed that tirofiban conferred a significant benefit in patients with BAD (aOR 0.44, 95% CI 0.20–0.97, *p* = 0.041), as illustrated in Figure 3. Additionally, a slight trend toward greater efficacy was observed in the tirofiban group among subpopulations including male patients, individuals younger than 60 years, patients with symptom onset between 24 and 48 h, and those with large artery atherosclerosis; however, these tendencies did not attain statistical significance (Figure 3).

## 4 Discussion

This multicenter prospective cohort study demonstrated that early intravenous administration of tirofiban could effectively reduce in‐hospital END and improve early neurological function and 90d outcomes in AIS patients without LVO, with baseline NIHSS scores ranging from 4 to 15, who were assigned within 48 h after symptom onset, compared with oral antiplatelet therapy, as indicated by both multivariate regression analysis and PSM analysis. Moreover, tirofiban administration was not associated with an increased risk of either intracranial or extracranial hemorrhage.

In this study, an increase of ≥ 2 points in the NIHSS score within 7 days of admission was defined as the criterion for END evaluation. The overall incidence rate of in‐hospital END was observed to be 13.7%, consistent with findings from previous studies [2, 3, 19]. However, due to the absence of data on the initial disease status of patients, this incidence rate did not account for those who might have already experienced END before admission. Therefore, the actual incidence of END in real‐world clinical settings may be higher than observed. The findings of this study showed that tirofiban significantly reduced the occurrence of in‐hospital END among AIS patients, in line with recent randomized controlled trial evidence [12, 19]. Different from most previous studies that focused on a 24‐h treatment window for tirofiban administration following symptom onset, our study extended the window to 48 h. The results of subgroup analysis indicated a promising trend in the efficacy of tirofiban among AIS patients treated within 24 to 48 h, suggesting its potential effectiveness over a broader time window. As a potent and fast‐acting GP IIb/IIIa receptor antagonist, tirofiban can block the final common pathway of platelet aggregation, thereby dissolving formed microthrombi, and inhibiting the formation of new thrombi, and stabilizing the ischemic penumbra. This may be the potential mechanism for tirofiban to achieve clinical benefits.

Regarding the role of tirofiban in improving neurological function prognosis, whether in the short term or long term, findings from previous studies have been inconsistent [12, 15, 17, 19, 29]. Based on its pharmacological mechanism, we hypothesize that tirofiban may demonstrate greater efficacy in patients with atherosclerotic AIS. Our study found that tirofiban significantly improves early or long‐term neurological function. Compared with previous studies, this study excluded patients with cardiogenic embolism, enrolled a larger cohort of patients with atherosclerotic cerebral infarction, and effectively reduced the incidence of END, which may account for the more pronounced therapeutic effect.

The present study revealed a notable observation that tirofiban showed a potential increased efficacy in patients with BAD, as indicated by the sub‐analysis. Previous studies have reported that the prevalence of BAD among Asian stroke patients is approximately 20.4% [30], with most cases presenting as moderate strokes. In this study, patients meeting the criteria for BAD accounted for 55% of the total sample. This higher proportion may be attributed to the exclusion of AIS with LVO in this study. Prior studies have highlighted a strong association between perforator artery occlusive stroke and END [31, 32]. However, the underlying pathogenesis of BAD remains unclear, although it is frequently associated with atherosclerotic plaques [33, 34]. Currently, evidence supporting the use of tirofiban in BAD is limited to studies with small sample sizes. Due to inconsistent definitions of BAD, our study relied on post hoc judgment based on Non‐contrast MRI scan to identify BAD, without employing high‐resolution MRI to assess vascular wall pathology and clarify the underlying etiology. Therefore, our findings represented a preliminary exploration, and future studies should incorporate large‐sample randomized controlled trials to further evaluate the potential therapeutic benefits of tirofiban in patients with BAD.

In terms of safety, our study showed that tirofiban did not significantly increase the risk of sICH or systemic bleeding and could be safely administered within an extended time window. However, the RESCUE BT2 study reported a higher incidence of sICH in the tirofiban group compared to oral antiplatelet therapy [12]. This discrepancy may be attributed to the inclusion of patients with more severe neurological deficits in the RESCUE BT2 study, as indicated by a median NIHSS score of 9 points. Additionally, the RESCUE BT2 study included patients whose symptoms did not improve within 24 h after IVT. In contrast, this study excluded patients who had received IVT treatment, and the baseline NIHSS score was lower, with a median of 5 points, resulting in a relatively lower overall bleeding risk. The ASSET study, recently presented at the European Stroke Organization Conference, assessed the efficacy and safety of tirofiban administered within 1 h after IVT in improving functional outcomes. The study findings indicated no statistically significant difference in the rate of sICH between the tirofiban and placebo groups, further supporting its safety profile in the treatment of AIS.

This study has several limitations. Firstly, this controlled study was non‐blinded and non‐randomized in design. While dual assessment was implemented during the evaluation process to minimize assessment bias and multiple logistic regression was used to adjust for potential confounding factors, selection bias cannot be entirely ruled out. Secondly, the relatively small sample size and lower baseline NIHSS scores may have underestimated the bleeding risk associated with tirofiban. Thirdly, this study excluded AIS patients with mild stroke (NIHSS < 4), patients with severe stroke (NIHSS > 15), and patients with LVO. These exclusion criteria may, to some extent, limit the generalizability of the study findings. Fourth, the diagnosis of BAD is not routinely verified through high‐resolution magnetic resonance vessel wall imaging, which may introduce certain uncertainties regarding the accuracy of BAD classification. Therefore, additional large‐sample randomized controlled trials are needed to further validate the findings of this study.

## 5 Conclusion

Compared to oral antiplatelet therapy, early intravenous administration of tirofiban can effectively prevent END and improve early neurological outcomes in AIS patients without LVO, and with no concomitant increase in bleeding risk, who were assigned within 48 h after symptom onset. Extending the tirofiban administration time window up to 48 h after symptom onset may be feasible. The potential application of tirofiban in patients with BAD holds promise and warrants future investigation.

## Funding

The study was funded by Dongguan Science and Technology Bureau (20231800905272), Medical Joint Fund of Jinan University (YXJC2022010), Science and Technology Projects in Guangzhou (2025A03J4094 and 2024A03J0816), National Natural Science Foundation of China (82571643), Guangdong Basic and Applied Basic Research Foundation (2023A1515010218), Medical Science and Technology Research of Guangdong Province (A2023136).

## Ethics Statement

The study was reviewed and approved by the ethics committee of the Sixth Affiliated Hospital of Jinan University (MEC [2022] 021). All clinical and laboratory procedures were conducted in accordance with the ethical principles outlined in the Declaration of Helsinki. Written informed consent was obtained from each participant or their legally authorized representative prior to enrollment.

## Conflicts of Interest

The authors declare no conflicts of interest.

## Data Availability

The data that support the findings of this study are available from the corresponding author upon reasonable request.

## References

[1] J. Kwan and P. Hand , “Early Neurological Deterioration in Acute Stroke: Clinical Characteristics and Impact on Outcome,” QJM : An International Journal of Medicine 99 (2006): 625–633. 16905751 10.1093/qjmed/hcl082

[2] B. Indredavik , G. Rohweder , E. Naalsund , and S. Lydersen , “Medical Complications in a Comprehensive Stroke Unit and an Early Supported Discharge Service,” Stroke 39 (2008): 414–420. 18096834 10.1161/STROKEAHA.107.489294

[3] C. Weimar , T. Mieck , J. Buchthal , C. E. Ehrenfeld , E. Schmid , and H.‐C. Diener , “Neurologic Worsening During the Acute Phase of Ischemic Stroke,” Archives of Neurology 62 (2005): 393–397. 15767504 10.1001/archneur.62.3.393

[4] Q. Ye , F. Zhai , B. Chao , et al., “Rates of Intravenous Thrombolysis and Endovascular Therapy for Acute Ischaemic Stroke in China Between 2019 and 2020,” Lancet Regional Health 21 (2022): 100406. 10.1016/j.lanwpc.2022.100406 PMC8873940 35243459

[5] W. J. Powers , A. A. Rabinstein , T. Ackerson , et al., “Guidelines for the Early Management of Patients With Acute Ischemic Stroke: 2019 Update to the 2018 Guidelines for the Early Management of Acute Ischemic Stroke: A Guideline for Healthcare Professionals From the American Heart Association/American Stroke Association,” Stroke 50 (2019): e344–e418. 31662037 10.1161/STR.0000000000000211

[6] M. Yang , X. Huo , Z. Miao , and Y. Wang , “Platelet Glycoprotein IIb/IIIa Receptor Inhibitor Tirofiban in Acute Ischemic Stroke,” Drugs 79 (2019): 515–529. 30838514 10.1007/s40265-019-01078-0

[7] M. Schwarz , G. Meade , P. Stoll , et al., “Conformation‐Specific Blockade of the Integrin GPIIb/IIIa: A Novel Antiplatelet Strategy That Selectively Targets Activated Platelets,” Circulation Research 99 (2006): 25–33. 16778135 10.1161/01.RES.0000232317.84122.0c

[8] C. P. Cannon , W. S. Weintraub , L. A. Demopoulos , et al., “Comparison of Early Invasive and Conservative Strategies in Patients With Unstable Coronary Syndromes Treated With the Glycoprotein IIb/IIIa Inhibitor Tirofiban,” New England Journal of Medicine 344 (2001): 1879–1887. 11419424 10.1056/NEJM200106213442501

[9] A. W. J. Van't Hof , J. Ten Berg , T. Heestermans , et al., “Prehospital Initiation of Tirofiban in Patients With ST‐Elevation Myocardial Infarction Undergoing Primary Angioplasty (On‐TIME 2): A Multicentre, Double‐Blind, Randomised Controlled Trial,” Lancet 372 (2008): 537–546. 18707985 10.1016/S0140-6736(08)61235-0

[10] S. Windecker , P. Kolh , F. Alfonso , et al., “2014 ESC/EACTS Guidelines on Myocardial Revascularization: The Task Force on Myocardial Revascularization of the European Society of Cardiology (ESC) and the European Association for Cardio‐Thoracic Surgery (EACTS)developed With the Special Contribution of the European Association of Percutaneous Cardiovascular Interventions (EAPCI),” European Heart Journal 35 (2014): 2541–2619. 25173339 10.1093/eurheartj/ehu278

[11] F. A. Cura , D. L. Bhatt , A. M. Lincoff , et al., “Pronounced Benefit of Coronary Stenting and Adjunctive Platelet Glycoprotein IIb/IIIa Inhibition in Complex Atherosclerotic Lesions,” Circulation 102 (2000): 28–34. 10880411 10.1161/01.cir.102.1.28

[12] W. Zi , J. Song , W. Kong , et al., “Tirofiban for Stroke Without Large or Medium‐Sized Vessel Occlusion,” New England Journal of Medicine 388 (2023): 2025–2036. 37256974 10.1056/NEJMoa2214299

[13] Y. Du , Y. Li , Z. Duan , et al., “The Efficacy and Safety of Intravenous Tirofiban in the Treatment of Acute Ischemic Stroke Patients With Early Neurological Deterioration,” Journal of Clinical Pharmacy and Therapeutics 47 (2022): 2350–2359. 36461632 10.1111/jcpt.13816

[14] H. Sang , Z. Cao , J. Du , et al., “Intravenous Tirofiban Versus Alteplase Before Endovascular Treatment in Acute Ischemic Stroke: A Pooled Analysis of the DEVT and RESCUE BT Trials,” Stroke 55 (2024): 856–865. 38362756 10.1161/STROKEAHA.123.044562

[15] Z. Qiu , F. Li , H. Sang , et al., “Effect of Intravenous Tirofiban vs Placebo Before Endovascular Thrombectomy on Functional Outcomes in Large Vessel Occlusion Stroke: The RESCUE BT Randomized Clinical Trial,” Journal of the American Medical Association 328 (2022): 543–553. 35943471 10.1001/jama.2022.12584 PMC9364124

[16] B. Han , T. Ma , Z. Liu , et al., “Efficacy and Safety of Tirofiban in Clinical Patients With Acute Ischemic Stroke,” Frontiers in Neurology 12 (2021): 785836. 35211073 10.3389/fneur.2021.785836 PMC8860815

[17] C. Wu , C. Sun , L. Wang , et al., “Low‐Dose Tirofiban Treatment Improves Neurological Deterioration Outcome After Intravenous Thrombolysis,” Stroke 50 (2019): 3481–3487. 31570084 10.1161/STROKEAHA.119.026240

[18] W. Zhao , R. Che , S. Shang , et al., “Low‐Dose Tirofiban Improves Functional Outcome in Acute Ischemic Stroke Patients Treated With Endovascular Thrombectomy,” Stroke 48 (2017): 3289–3294. 29127270 10.1161/STROKEAHA.117.019193

[19] W. Zhao , S. Li , C. Li , et al., “Effects of Tirofiban on Neurological Deterioration in Patients With Acute Ischemic Stroke: A Randomized Clinical Trial,” JAMA Neurology 81 (2024): 594–602. 38648030 10.1001/jamaneurol.2024.0868 PMC11036313

[20] Investigators P‐PS , “Inhibition of the Platelet Glycoprotein IIb/IIIa Receptor With Tirofiban in Unstable Angina and Non‐Q‐Wave Myocardial Infarction,” New England Journal of Medicine 338 (1998): 1488–1497. 9599103 10.1056/NEJM199805213382102

[21] L. Liu , W. Chen , H. Zhou , et al., “Chinese Stroke Association Guidelines for Clinical Management of Cerebrovascular Disorders: Executive Summary and 2019 Update of Clinical Management of Ischaemic Cerebrovascular Diseases,” Stroke and Vascular Neurology 5 (2020): 159–176. 32561535 10.1136/svn-2020-000378 PMC7337371

[22] Neurology CMABo , “Chinese Guidelines for the Diagnosis and Treatment of Acute Ischemic Stroke 2023,” Chinese Journal of Neurology 57, no. 06 (2024): 523–559.

[23] H.‐M. Kwon , Y.‐S. Lee , H.‐J. Bae , and D.‐W. Kang , “Homocysteine as a Predictor of Early Neurological Deterioration in Acute Ischemic Stroke,” Stroke 45 (2014): 871–873. 24448992 10.1161/STROKEAHA.113.004099

[24] J.‐M. Kim , J.‐H. Bae , K.‐Y. Park , et al., “Incidence and Mechanism of Early Neurological Deterioration After Endovascular Thrombectomy,” Journal of Neurology 266 (2019): 609–615. 30631916 10.1007/s00415-018-09173-0

[25] P. Khatri , D. O. Kleindorfer , S. D. Yeatts , et al., “Strokes With Minor Symptoms: An Exploratory Analysis of the National Institute of Neurological Disorders and Stroke Recombinant Tissue Plasminogen Activator Trials,” Stroke 41 (2010): 2581–2586. 20814000 10.1161/STROKEAHA.110.593632 PMC2964419

[26] U. Neuberger , M. A. Möhlenbruch , C. Herweh , C. Ulfert , M. Bendszus , and J. Pfaff , “Classification of Bleeding Events: Comparison of ECASS III (European Cooperative Acute Stroke Study) and the New Heidelberg Bleeding Classification,” Stroke 48 (2017): 1983–1985. 28455322 10.1161/STROKEAHA.117.016735

[27] Y. Xu , Y. Chen , R. Chen , F. Zhao , P. Wang , and S. Yu , “External Validation of the WORSEN Score for Prediction the Deterioration of Acute Ischemic Stroke in a Chinese Population,” Frontiers in Neurology 11 (2020): 482. 32547483 10.3389/fneur.2020.00482 PMC7272667

[28] X. Men , W. Chen , Y. Xu , et al., “Chinese Expert Consensus on Branch Atheromatous Disease,” Chinese Journal of Stroke 16 (2021): 508–514.

[29] G. Torgano , B. Zecca , V. Monzani , et al., “Effect of Intravenous Tirofiban and Aspirin in Reducing Short‐Term and Long‐Term Neurologic Deficit in Patients With Ischemic Stroke: A Double‐Blind Randomized Trial,” Cerebrovascular Diseases 29 (2010): 275–281. 20090319 10.1159/000275503

[30] B. J. Kim and J. S. Kim , “Ischemic Stroke Subtype Classification: An Asian Viewpoint,” Journal of Stroke 16 (2014): 8–17. 24741560 10.5853/jos.2014.16.1.8 PMC3961817

[31] W. Li , Y. Wu , X.‐S. Li , et al., “Intravenous Tirofiban Therapy for Patients With Capsular Warning Syndrome,” Stroke and Vascular Neurology 4 (2019): 22–27. 31105975 10.1136/svn-2018-000163 PMC6475082

[32] B. Liu , H. Zhang , R. Wang , et al., “Early Administration of Tirofiban After Urokinase‐Mediated Intravenous Thrombolysis Reduces Early Neurological Deterioration in Patients With Branch Atheromatous Disease,” Journal of International Medical Research 48 (2020): 300060520926298. 32459110 10.1177/0300060520926298 PMC7273788

[33] L. R. Caplan , “Intracranial Branch Atheromatous Disease: A Neglected, Understudied, and Underused Concept,” Neurology 39 (1989): 1246–1250. 2671793 10.1212/wnl.39.9.1246

[34] L. Petrone , S. Nannoni , A. Del Bene , V. Palumbo , and D. Inzitari , “Branch Atheromatous Disease: A Clinically Meaningful, Yet Unproven Concept,” Cerebrovascular Diseases 41 (2016): 87–95. 26671513 10.1159/000442577

